# Cryptochrome 1 and phytochrome B control shade-avoidance responses in Arabidopsis via partially independent hormonal cascades

**DOI:** 10.1111/j.1365-313X.2011.04598.x

**Published:** 2011-05-25

**Authors:** Mercedes M Keller, Yvon Jaillais, Ullas V Pedmale, Javier E Moreno, Joanne Chory, Carlos L Ballaré

**Affiliations:** 1 Ifeva, Consejo Nacional de Investigaciones Científicas y Técnicas, and Universidad de Buenos Aires, Avenida San Martín 4453 C1417DSE Buenos AiresArgentina; 2 Plant Biology Laboratory, Howard Hughes Medical Institute, The Salk Institute for Biological StudiesLa Jolla, CA 92037, USA

**Keywords:** blue light, brassinosteroid, phytochrome interacting factors (PIFs), PIN3, Tryptophan Aminotransferase of Arabidopsis 1, DELLA

## Abstract

Plants respond to a reduction in the red/far-red ratio (R:FR) of light, caused by the proximity of other plants, by initiating morphological changes that improve light capture. In Arabidopsis, this response (shade avoidance syndrome, SAS) is controlled by phytochromes (particularly phyB), and is dependent on the TAA1 pathway of auxin biosynthesis. However, when grown in real canopies, we found that *phyB* mutants and mutants deficient in TAAI (*sav3*) still display robust SAS responses to increased planting density and leaf shading. The SAS morphology (leaf hyponasty and reduced lamina/petiole ratio) could be phenocopied by exposing plants to blue light attenuation. These responses to blue light attenuation required the UV-A/blue light photoreceptor cry1. Moreover, they were mediated through mechanisms that showed only limited overlap with the pathways recruited by phyB inactivation. In particular, pathways for polar auxin transport, auxin biosynthesis and gibberellin signaling that are involved in SAS responses to low R:FR were not required for the SAS responses to blue light depletion. By contrast, the brassinosteroid response appeared to be required for the full expression of the SAS phenotype under low blue light. The phyB and cry1 inactivation pathways appeared to converge in their requirement for the basic/helix-loop-helix (bHLH) transcription factors PHYTOCHROME INTERACTING FACTORs 4 and 5 (PIF4 and PIF5) to elicit the SAS phenotype. Our results suggest that blue light is an important control of SAS responses, and that PIF4 and PIF5 are critical hubs for a diverse array of signaling routes that control plant architecture in canopies.

## Introduction

Competition is a critical determinant of plant fitness in dense populations. One strategy used by plants to improve their competitive success is based on morphological plasticity, where the shape of the plant body is constantly remodeled to optimize the capture of light and other resources (Aphalo and Ballaré, 1995; Ballaré, 1999; Dorn *et al.*, 2000; De Kroon *et al.*, 2009; Novoplansky, 2009; Sultan, 2010). A prime example of morphological plasticity is the shade avoidance syndrome (SAS) (Smith, 1982). SAS responses typically include increased elongation of the stem and petioles, leaf hyponasty, reduced branching and phototropic orientation of the plant shoot towards gaps in the canopy (Ballaré, 1999).

The SAS responses are triggered and controlled by multiple canopy signals, particularly signals in the light environment (reviewed in Ballaré, 1999; Vandenbussche *et al.*, 2005; Ballaré, 2009; Keuskamp *et al.*, 2010b). The best characterized of these signals is the red/far red ratio (R:FR; 660–670/725–735 nm), which decreases in response to canopy density because of the strong absorption of red light by chlorophyll and scattering of far-red photons by cell walls and other plant constituents. Other potentially important light signals controlling SAS responses include variations in total irradiance, and specific changes in the blue light component caused by the absorption of visible wavelengths by chlorophyll and other leaf pigments (reviewed in Ballaré, 2009; Keuskamp *et al.*, 2010b).

Phytochrome B (phyB) is the major photoreceptor that senses a reduction in the R:FR ratio, and controls the initial appearance of SAS phenotypes (reviewed in Franklin, 2008; Ballaré, 2009; Jaillais and Chory, 2010; Martínez-García *et al.*, 2010). In fully de-etiolated Arabidopsis plants in the rosette stage, SAS responses to low R:FR (increased petiole elongation and leaf hyponasty) depend on increased auxin biosynthesis through the TRYPTOPHAN AMINOTRANSFERASE OF ARABIDOPSIS 1 (TAA1) pathway (Tao *et al.*, 2008; Moreno *et al.*, 2009), and polar auxin transport by the auxin efflux carrier PIN3 (Keuskamp *et al.*, 2010a). Increased gibberellin (GA) production, perhaps in response to increased auxin (Frigerio *et al.*, 2006), is also required for the expression of petiole elongation responses to low R:FR in Arabidopsis plants at the rosette stage (Djakovic-Petrovic *et al.*, 2007). Increased GA production triggers degradation by the 26S proteasome of DELLA proteins, a group of five nuclear proteins that redundantly repress growth (Djakovic-Petrovic *et al.*, 2007). DELLA proteins bind to and inactivate PHYTOCHROME INTERACTING FACTORS (PIFs) (Feng *et al.*, 2008; de Lucas *et al.*, 2008), which are growth-promoting transcription factors involved in the elicitation of SAS responses to low R:FR (Lorrain *et al.*, 2008; Hornitschek *et al.*, 2009). Brassinosteroids (BRs) (Kozuka *et al.*, 2010) and ethylene (Pierik *et al.*, 2009) are additional hormones that have been implicated as playing a role during the elicitation of SAS responses to low R:FR ratios.

A reduction in blue light fluence rate may also provide plants with information on the proximity of competitors, and trigger adaptive SAS responses. Blue light inhibits hypocotyl growth in recently germinated seedlings, and this effect is mediated by the cryptochrome photoreceptors (Cashmore *et al.*, 1999; Folta and Spalding, 2001; Pierik *et al.*, 2009; Sellaro *et al.*, 2010). Blue light gradients can drive phototropic responses of seedlings in patchy canopies (Ballaré*et al.*, 1992), an effect probably mediated by the phototropins (Briggs and Christie, 2002; Takemiya *et al.*, 2005). Even fully de-etiolated plants can present strong SAS-like responses to blue light attenuation, including increased main-stem elongation (Ballaré*et al.*, 1991), petiole elongation (Kozuka *et al.*, 2005) and leaf hyponasty (Pierik *et al.*, 2004; Millenaar *et al.*, 2009).

The importance of blue light signals in the control of morphological plasticity in plant canopies has not been clearly established. Furthermore, it is not known whether the hormone signaling circuits triggered by depletion of the active form of phyB (Pfr) in response to low R:FR are also recruited to elicit SAS responses to blue light attenuation. These questions are addressed in this article, using a combination of canopy and physiological experiments with Arabidopsis plants at the rosette stage. We evaluated variations in leaf morphology (lamina/petiole length ratio, L:P) and leaf angle (hyponasty) as the principal indicators of SAS. We found that mutants deficient in R:FR responses show robust morphological responses to increased canopy density or leaf shading, suggesting that additional light-regulated pathways are involved in SAS. The high-density/shade morphology could be phenocopied by exposing plants to light in which the blue light component was attenuated. These blue light responses required cry1, and were mediated through hormonal pathways that showed only limited overlap with the pathways activated in response to phyB Pfr depletion by low R:FR ratios. Interestingly, our results demonstrate that PIF4 and PIF5, which are basic/helix-loop-helix (bHLH) transcription factors known to mediate morphological responses to phyB inactivation, are also required for the elicitation of the SAS phenotype in response to blue light attenuation.

## Results

### Mutants deficient in R:FR responses display SAS-like responses in canopies

**Figure 1:**
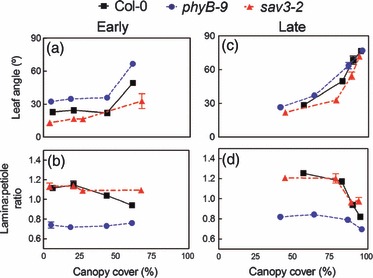
The *sav3* and *phyB* mutations reduce the shade avoidance syndrome (SAS) responses to crowding in low, but not in high, levels of canopy cover. Variations in leaf angle and lamina/petiole length (L:P) of Arabidopsis plants were measured in response to crowding (increased canopy density) in a density gradient experiment. The experiment was carried out in a glasshouse, under natural radiation during the autumn in Buenos Aires (see Experimental procedures for details). There were four trays for each genotype and canopy density combination; the nine central plants were measured in each tray. Bars indicate ±1 SE (*n* = 4). (a, b) Measurements of leaf angle and leaf morphology 25 days after germination. (c–d) Measurements of leaf angle and leaf morphology 45 days after germination.

Phytochrome B and increased auxin biosynthesis through the TAA1 pathway are essential for the response of plants to variations in the R:FR ratio caused by the proximity of other plants (Tao *et al.*, 2008; Moreno *et al.*, 2009). We tested the effects of the *phyB-9* and *sav3-2* mutations, which affect the genes encoding for the PHYB apoprotein and the TAA1 enzyme, respectively, on the responses of Arabidopsis plants to real plant neighbors in a series of glasshouse experiments under the full solar spectrum. When wild-type (Col-0) Arabidopsis plants were grown in monocultures of different densities they responded to crowding with characteristic SAS responses (Figure 1). These responses included increased leaf angles (hyponasty) and reduced growth of the leaf lamina relative to the petiole (reduced L:P ratio). Compared with Col-0 plants, *phyB* mutants had constitutively hyponastic leaves and low L:P ratios. *sav3-2* plants showed significantly attenuated morphological responses to crowding, but only during the early part of the experiment, when the canopy cover was small (Figure 1a,b; day 25). As the plants grew bigger and the canopies closed (greater canopy cover), both *sav3-2* and *phyB* plants displayed clear morphological responses to increased levels of crowding (Figure 1c,d; day 45).

**Figure 2:**
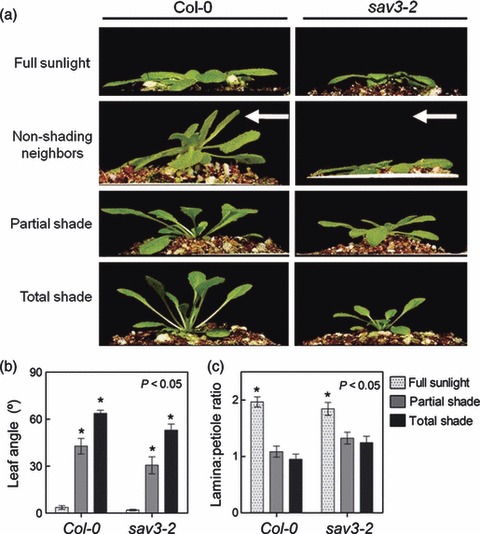
The *sav3* mutation eliminates shade avoidance syndrome (SAS) responses to the proximity of non-shading neighbors, but does not prevent SAS responses to canopy shading. Plants were grown in a glasshouse under natural radiation under the following conditions: with no neighbors (full sunlight); on the north side of a thick ryegrass canopy (non-shading neighbors); or under the ryegrass canopy and two levels of leaf shading (partial shade and total shade). The experiment was carried out during the autumn in Buenos Aires (see Experimental procedures for details). (a) Representative photographs of the rosettes taken after 7 days of treatment. The arrow in the second row indicates the predominant direction of the radiation reflected from the grass leaves, and phototropic bending away from this radiation in Col-0, but not in *sav3-2.* (b and c) Quantitative data for the SAS response in the two shading treatments. Thin bars indicate ±1 SE (*n* = 6–10). *Significant effect (*P* < 0.05).

Neighbor proximity was further manipulated by growing Arabidopsis plants in front of or underneath canopies of annual ryegrass (*Lolium multiflorum*). Rosettes of the Col-0 plants responded to FR radiation reflected from the grass canopy with a typical SAS phenotype, even though the canopy did not shade the target plants; this response to reflected FR was missing in *sav3-2* (Figure 2a, row 2; non-shading neighbors). In contrast, when plants were placed under the ryegrass canopy and were actually shaded by the grass leaves, Col-0 and the *sav3-2* mutant displayed very similar SAS responses (Figure 2a, rows 3 and 4; see quantitative data in Figure 2b,c).

### SAS responses to blue light attenuation are retained in *sav3* and *pin3* mutants

It is well established that reflected FR is the principal signal of canopy density in open canopies (with a low leaf area index, LAI), but at high LAIs other signals are thought to play an important role, including the level of blue light received per plant, which decreases as the density of the canopy increases (references in Ballaré, 1999). Therefore, we reasoned that the responses of *sav3-2* to crowding under high levels of cover (Figure 1), and to shading by the ryegrass canopy (Figure 2), could reflect a conserved response to blue light attenuation.

**Figure 3:**
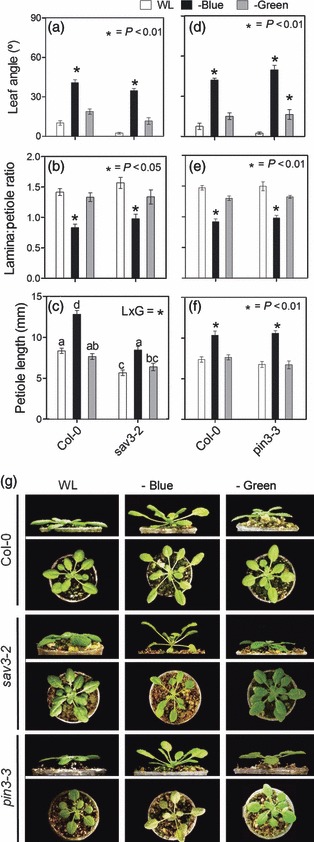
Arabidopsis plants at the rosette stage show robust shade avoidance syndrome (SAS) responses to blue light attenuation, which are conserved in *sav3* and *pin3* mutants. Plants were grown under white light (WL) or WL filtered through a yellow filter (–BLUE) or a pink filter (–GREEN), which was used as a control for the PAR reduction caused by blue light attenuation. Morphological measurements and photographs were taken after 7 days of treatment. Thin bars indicate ±1 SE (*n* = 10–16 individual plants). *Significant effects of the light treatments at the indicated *P* values. Different letters indicate significant differences between means in cases in which the light × genotype interaction term (L × G) was significant. (a–c) Comparison of morphological responses between Col-0 and *sav3* plants. (d–f) Comparison of morphological responses between Col-0 and *pin3* plants. (g) Representative photographs of the plants after exposure to the indicated light treatments.

We directly tested the effect of blue light attenuation on plants at the rosette stage, similar to those used in the canopy experiments described above, by using selective light filters. Plants responded to blue light attenuation with strong hyponasty and reduced L:P ratios (Figure 3). The SAS response to blue light attenuation was probably mediated by a specific blue light photoreceptor, as we found little or no response to green light attenuation, when a control filter was used to test for the effects of reducing photosynthetically active radiation (PAR). Importantly, the SAS responses to blue light depletion were clearly conserved in the *sav3-2* mutant (Figure 3a–c,g).

Recent work showed that in addition to TAA1 the auxin efflux carrier PIN3 is required for petiole elongation triggered by low R:FR ratios in rosette-stage plants (Keuskamp *et al.*, 2010a). This suggests that auxin produced in the leaves by TAA1 in response to low R:FR is actively transported to its site of action by PIN3. However, in the case of SAS responses elicited by blue light attenuation, we found a completely wild-type response in the *pin-formed3* (*pin3-3*) mutant (Figure 3d–f,h).

Next, we addressed whether other auxin biosynthesis or transport pathways are involved in blue light attenuation responses in rosette plants. First, we tested the requirement for auxin biosynthesis catalysed by YUCCA proteins. YUCCA proteins are flavin monooxygenases, and form a family of 11 members in Arabidopsis (Zhao *et al.*, 2001). A quintuple *yucca* mutant (lacking yuc3, yuc5, yuc7, yuc8 and yuc9; Tao *et al.*, 2008) displayed normal responses to blue light attenuation (Figure S1). We also tested the effect of 1-N-naphthylphthalmic acid (NPA), an inhibitor of polar auxin transport (Okada *et al.*, 1991), on SAS responses to attenuated blue light. The NPA experiments produced results that were difficult to interpret, because, as found in previous studies (reviewed in van Zanten *et al.*, 2010), low doses of NPA per se induced leaf hyponasty in the control plants grown under white light (Figure S2). For the other SAS responses characterized in our experiments (reduction of L:P ratio), 5 μm NPA tended to cancel the effect of blue light depletion, mainly by reducing petiole elongation (Figure S2). These results suggest that polar auxin transport may play a role in the activation of the SAS response to blue light attenuation, but the auxin biosynthesis and transport genes that are critical for activating the SAS response to low R:FR (*SAV3* and *PIN3*) are not essential for blue light responses.

### Blue light attenuation does not induce DELLA turnover in rosette plants

**Figure 4:**
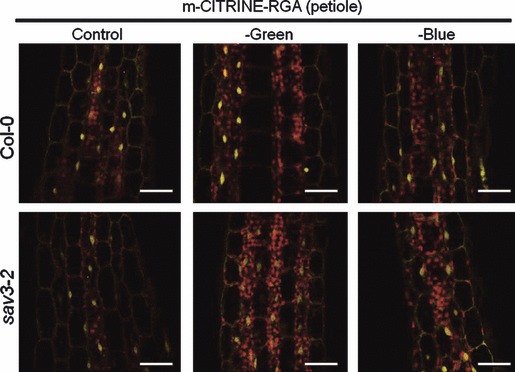
Blue light attenuation fails to trigger DELLA degradation in Arabidopsis petioles. Plants were grown for 2 weeks under white light and then exposed to the indicated light treatments for 7 days. Control, clear polyester; –green, pink filter; –blue, yellow filter. Scale bars: 25 μm.

Petiole elongation responses induced by low R:FR in mature plants correlate with DELLA turnover induced by gibberellins, and are eliminated by enhanced DELLA stability in the gain-of-function *gai-1* mutant (Djakovic-Petrovic *et al.*, 2007). Therefore, we wanted to determine whether the blue light attenuation effects inducing SAS responses are associated with increased DELLA degradation. We tested the effect of blue light attenuation on DELLA stability using transgenic lines expressing RGA fused to the fluorescent protein mCITRINE at its N terminus. To circumvent the potential transcriptional regulation of *RGA* by blue light attenuation, we used the mild constitutive promoter of the *POLYUBIQUITIN10* gene (*pUBQ10*). Our *pUBQ10::mCITRINE-RGA* line did not harbor any developmental phenotypes (data not shown), and GA treatment triggered rapid mCITRINE-RGA turnover (Figure S3). We found that blue light attenuation treatments that are highly effective at inducing the SAS phenotype (Figure 3) failed to increase DELLA degradation in both Col-0 and the *sav3* mutant (Figure 4). These results indicate that blue light attenuation and phyB inactivation by low R:FR have very different effects on DELLA turnover in Arabidopsis plants at the rosette stage.

**Figure 5:**
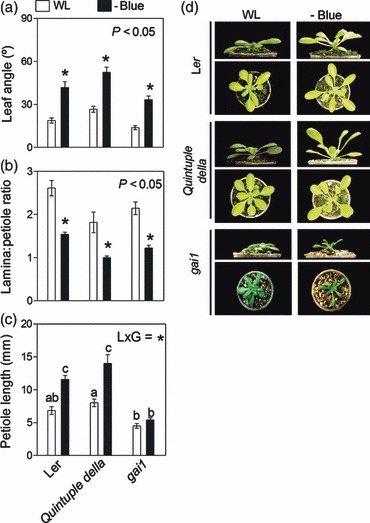
Shade avoidance syndrome (SAS) responses of Arabidopsis rosettes to blue light attenuation are conserved in global *della* mutants and in the *gai-1* gain-of-function mutant, which is impaired in DELLA turnover. Plants were grown under white light (WL) or WL filtered through a yellow filter (–BLUE). Morphological measurements and photographs were taken after 7 days of treatment. Thin bars indicate ±1 SE (*n* = 8–12 individual plants). *Significant effects of light treatment at the indicated *P* values. Different letters indicate significant differences between means in cases in which the light × genotype interaction term (L × G) was significant. (a–c) Comparison of morphological responses between L*er* and the mutants impaired in DELLA function. (d) Representative photographs of the plants after exposure to the indicated light treatments.

We directly tested the role of DELLA turnover in SAS responses induced by blue light attenuation by using a quintuple (global) *della* mutant (Feng *et al.*, 2008) and the *gai-1* gain-of-function mutant (Koorneef *et al.*, 1985), which is impaired in DELLA degradation (all in the Landsberg *erecta* background, L*er*). L*er* plants are known to be less responsive than those of the Col-0 accession in terms of their hyponastic response, when the response is induced by ethylene (Benschop *et al.*, 2007). However, in our filter experiments, L*er* plants showed a clear hyponastic response to blue light attenuation (Figure 5), which is consistent with the idea that ethylene and light attenuation trigger hyponastic responses through independent pathways (Millenaar *et al.*, 2009). The global *della* mutant clearly retained the capacity to respond to blue light attenuation with a typical SAS repertoire (leaf hyponasty and reduced L:P ratio) (Figure 5). As expected, plants of the *gai-1* mutant were significantly dwarfed compared with the L*er* wild type (Koorneef *et al.*, 1985). This phenotype is consistent with the persistence of the growth inhibitory effects of the stable version of the GAI protein (Harberd *et al.*, 2009). However, the effects of blue light depletion promoting leaf hyponasty and reducing the L:P ratio were retained, although the expression of the response was not as intense as in the L*er* wild type. Absolute petiole elongation was not promoted by low blue light in *gai-1* (Figure 5c). Taken together, these results indicate that DELLA turnover is neither induced by blue light attenuation (Figure 4) nor directly involved in triggering specific growth remodeling components of the SAS phenotype (hyponasty and reduced L:P ratio) induced by blue light attenuation (Figure 5). Nevertheless, abnormal DELLA stability may reduce the L:P response, probably as a consequence of persistent inhibition of petiole elongation (Figure 5c).

### Brassinosteroid signaling is required for elongation responses to blue light depletion

**Figure 6:**
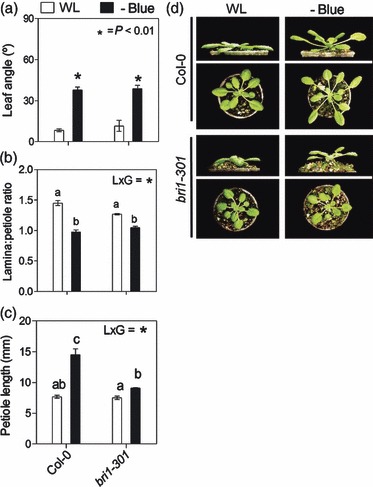
Shade avoidance syndrome (SAS) responses of Arabidopsis rosettes to blue light attenuation are partially dependent on brassinosteroid (BR) sensitivity. Plants were grown under white light (WL) or WL filtered through a yellow filter (–BLUE). Morphological measurements and photographs were taken after 7 days of treatment. Thin bars indicate ±1 SE (*n* = 8–12 individual plants). *Significant effects of light treatment at the indicated *P* values. Different letters indicate significant differences between means in cases in which the light × genotype interaction term (L × G) was significant. (a–c) Comparison of morphological responses between Col-0 and the *bri1-301* mutant. (d) Representative photographs of the plants after exposure to the indicated light treatments.

Studies with Arabidopsis plants at the rosette stage (Kozuka *et al.*, 2010) showed that BR (along with auxin) are involved in controlling petiole elongation and leaf morphology responses to end-of-day low R:FR treatments. Furthermore, studies with recently germinated Arabidopsis seedlings established that auxin (from the TAA1 pathway) and BR signaling are both required for the effects of blue light attenuation in the promotion of hypocotyl elongation (Keuskamp *et al.*, 2011). We tested the response to blue light attenuation at the rosette stage in mutants impaired in BR synthesis and perception. The *det2-1* plants (impaired in BR biosynthesis) have a strong phenotype and very compact rosettes. Their growth is severely impaired, and no morphological responses to blue light attenuation were evident in this mutant (Figure S4). Using a mutant that carries a weak allele of *BRASSINOSTEROID INSENSITIVE 1*, *bri1-301*, we found that the hyponastic response to blue light attenuation was completely retained. However, the effect of blue light attenuation reducing L:P ratio was impaired in *bri1-301*, mainly because petiole elongation was inhibited (Figure 6).

### SAS responses to blue light attenuation in rosette plants are probably mediated by cry1

**Figure 7:**
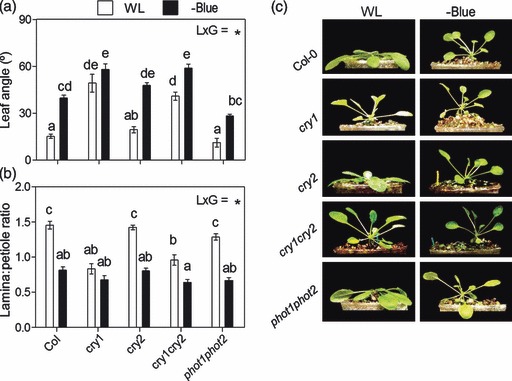
Cry1 is the photoreceptor that mediates SAS responses of rosette plants to blue light attenuation. Plants were grown under white light (WL) or WL filtered through a yellow filter (–BLUE). Morphological measurements and photographs were taken after 7 days of treatment. Thin bars indicate ±1 SE (*n* = 8–16 individual plants). The light × genotype interaction term (L × G) was significant in (a) and (b) (*P* < 0.01). Different letters indicate significant differences between means. (a, b) Morphological responses to blue light attenuation in Col-0 plants and in the various blue light photoreceptor mutants. (c) Representative photographs of the plants after exposure to the indicated light treatments.

Cryptochromes are known to be involved in the leaf hyponastic response of Arabidopsis rosettes to neutral shade (Millenaar *et al.*, 2009), and both cry1 and cry2 were found to be required for the inhibitory effect of monochromatic blue light on petiole elongation (Kozuka *et al.*, 2005). We tested blue light receptor mutants for their SAS responses to blue light attenuation. In the *cry1* single mutants and *cry1 cry2* double mutants the leaves were constitutively hyponastic, and had reduced L:P ratios compared with the Col wild type (Figure 7). *cry2* had a normal phenotype, whereas the *phot1 phot2* phototropin double mutant showed a slightly attenuated response in terms of hyponasty and normal L:P response (Figure 7). These results suggest that the SAS-like response to blue light attenuation in rosette plants of the Col-0 ecotype is principally mediated by cry1 inactivation. Differences among accessions may be present, however, as previous studies in the L*er* background did not report observations of hyponastic leaves for plants of the *cry1* single mutant under white light (Ballaré and Scopel, 1997; Mullen *et al.*, 2006).

### The response to blue light attenuation requires PIF4 or PIF5

**Figure 8:**
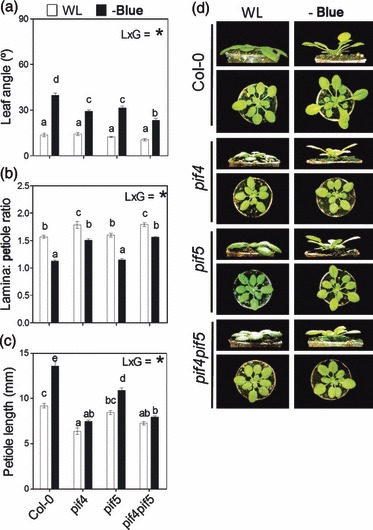
PIF4 and PIF5 are required for the full expression of the shade avoidance syndrome (SAS) phenotype to blue light attenuation. Plants were grown under white light (WL) or WL filtered through a yellow filter (–BLUE). Morphological measurements and photographs were taken after 7 days of treatment. Thin bars indicate ±1 SE (*n* = 8–20 individual plants). The light × genotype interaction term (L × G) was significant (*P* < 0.05) in all panels; different letters indicate significant differences between means. (a–c) Morphological responses to blue light attenuation in Col-0 plants and in the various *pif* mutants. (d) Representative photographs of the plants after exposure to the indicated light treatments.

In seedlings, hypocotyl elongation responses triggered by low R:FR (or the *phyB* mutation) are partially dependent on the bHLH transcription factors PIF4 and PIF5 (Lorrain *et al.*, 2008; Hornitschek *et al.*, 2009). We found that compared with Col-0 the hyponastic response to blue light attenuation was reduced in *pif4* and *pif5* single mutants, and even more so in the *pif4 pif5* double mutant (Figure 8). In terms of leaf morphology, *pif4* had a greater impact than *pif5* in reducing the L:P ratio and petiole elongation response to blue light attenuation (Figure 8). These results suggest that both of these bHLH transcription factors, but particularly PIF4, are required for the full activation of the SAS phenotype in response to low levels of blue light.

## Discussion

Plants perceive the proximity of neighboring plants using specific photoreceptors, which modulate the expression of growth and tropic responses, and allow the plant to forage for photons in heterogeneous light environments. A critical signal of neighbor proximity is a low R:FR ratio, which reduces the levels of the active form of phyB and other stable phytochromes, and leads to the appearance of SAS (reviewed in Franklin, 2008; Ballaré, 2009; Keuskamp *et al.*, 2010b). Two hallmarks of SAS in Arabidopsis and other rosette plants are leaf hyponasty and reduced growth of the leaf lamina relative to petiole growth (reduced L:P ratio). These responses help the plant to move its photosynthetic organs upwards and reach canopy strata with improved light conditions. In even-aged plant stands, both responses are triggered early on during canopy development, well before mutual shading among neighboring plants becomes intense. These responses are: (i) triggered by the low R:FR ratio of the light reflected by neighboring plants; (ii) mediated by the inactivation of phyB; and (iii) completely dependent on the TAA1 pathway of auxin biosynthesis (Tao *et al.*, 2008; Moreno *et al.*, 2009). In our canopy experiments, we found reduced SAS responses in the *sav3* mutant (compared with Col-0) under conditions of low canopy cover (Figure 1a,b) or in the proximity of non-shading neighbors (Figure 2a). In contrast, later in the density gradient experiment, when leaf cover was 70% or more (Figure 1c,d), or when the plants were placed underneath a dense grass canopy (Figure 2), *sav3* displayed robust hyponasty and L:P responses. Furthermore, even *phyB* plants showed exacerbation of their constitutively expressed SAS phenotype at high LAIs (Figure 1c,d). These observations demonstrate that phyB-independent light and hormonal signals participate in activating the SAS phenotype in Arabidopsis plants grown in real canopies.

A signal that may activate SAS responses to crowding or leaf shading is blue light attenuation, which results from the strong absorption of blue photons by chlorophyll (Ballaré*et al.*, 1991). Hypocotyl growth is inhibited by blue light (Cashmore *et al.*, 1999; Folta and Spalding, 2001; Pierik *et al.*, 2009; Sellaro *et al.*, 2010; Keuskamp *et al.*, 2011), and in fully de-etiolated plants grown under white light, blue light attenuation has been shown to cause increased internode elongation in mustard and *Datura ferox* (Ballaré*et al.*, 1991), and leaf hyponasty in *Nicotiana tabacum* (tobacco; Pierik *et al.*, 2004). Previous studies have shown that Arabidopsis plants at the rosette stage respond to treatments that reduce total light intensity with leaf hyponasty, and that this response can be partially reversed by reintroducing blue light in the low light treatment (Millenaar *et al.*, 2009). Our results confirm that Arabidopsis rosettes can display very marked SAS responses to blue light attenuation, including pronounced hyponasty and a reduced L:P ratio (Figure 3). Our data also suggest that, at least in Col-0, this response is predominantly mediated by cry1, with no obvious participation of cry2 or phototropins (Figure 7). Thus, cry1 is likely to participate in the SAS responses of Arabidopsis plants shaded by competitors (Figures 1 and 2).

The two SAS responses characterized in this study (hyponasty and altered leaf morphology), although concomitantly displayed in response to competition signals (low R:FR or low blue light), appeared to be controlled by different mechanisms. This difference between responses is apparent from the differential effects of the mutations tested in our experiments, which are summarized in Table 1. Therefore, L:P and leaf hyponasty responses will be considered separately in the discussion below.

**Table 1:** Summary of the effects of various mutations on the expression of different components of the shade avoidance syndrome (SAS) phenotype in response to blue light attenuation in rosette plants

Response	*cry1*	*sav3*	*pin3*	5 × *della*	*gai1*	*bri1*	*pif4*	*pif5*	*pif4 pif5*
Hyponasty	Constitutive	Normal	Normal	Normal	Reduced	Normal	Reduced	Reduced	Strongly reduced
Reduction of L:P	Constitutive	Normal	Normal	Normal	Normal	Reduced	Reduced	Normal	Reduced
Petiole elongation	Constitutive	Reduced	Normal	Normal	Strongly reduced	Strongly reduced	Strongly reduced	Reduced	Strongly reduced

L:P, lamina/petiole length ratio.

### Leaf morphology (L:P ratio)

Our results suggest that, at the rosette stage, the auxin and GA action pathways that are recruited by phyB inactivation are not engaged by cry1 inactivation, even though inactivation of both photoreceptors produce similar L:P phenotypes. The L:P response to blue light attenuation was completely independent of SAV3 and PIN3 (Figure 3). In contrast, SAV3 and PIN3 are required for the expression of leaf morphology responses elicited by low R:FR ratios in rosette plants (Tao *et al.*, 2008; Moreno *et al.*, 2009; Keuskamp *et al.*, 2010a), and are also known to play important roles in the hypocotyl elongation responses to low blue light and R:FR in young Arabidopsis seedlings (Tao *et al.*, 2008; Keuskamp *et al.*, 2010a, 2011). Moreover, whereas DELLA degradation appears to play a significant role in mediating SAS responses to low R:FR ratios in seedlings and rosette plants (Djakovic-Petrovic *et al.*, 2007), it was not activated by blue light attenuation (Figure 4), and not directly involved in the production of the SAS response, because blue light attenuation causes robust L:P responses in global *della* mutants (Figure 5). It is apparent, however, that the degradation of DELLA is somehow required for the full expression of the SAS phenotype in low levels of blue light, presumably because L:P responses and hyponasty ultimately depend on elongation, which is repressed in the mutants carrying stable DELLA proteins (*gai-1* in Figure 5c).

Brassinosteroids also appeared to be partially involved in the L:P response to blue light attenuation (Figure 6). Although BRs are known to participate in light-controlled signaling networks (Szekeres *et al.*, 1996; Neff *et al.*, 1999; Luccioni *et al.*, 2002; Luo *et al.*, 2010), very little is known about their involvement in SAS responses. A recent study (Kozuka *et al.*, 2010) demonstrated that BRs are required for petiole elongation and lamina growth inhibition responses to end-of-day FR pulses. Experiments reported in a companion paper (Keuskamp *et al.*, 2011) demonstrate that BRs are required, along with auxin, for the hypocotyl elongation response of Arabidopsis seedlings to blue light attenuation. Collectively, these studies suggest that BR may have a more important role than previously thought in the fine-tuning of SAS responses elicited by multiple canopy signals. The mechanism of BR action in SAS responses remains to be elucidated. The study of Keuskamp *et al.* (2011) suggests that, in Arabidopsis hypocotyls, BRs regulate elongation responses to blue light depletion via the regulation of specific sets of *XTH* genes.

### Leaf hyponasty

None of our experiments revealed a specific connection between the hyponastic response to blue light attenuation and hormonal action, as the leaf angle response to low blue light was present in all of the hormone-related mutants that we tested (Table 1). Furthermore, previous work has shown that ethylene, which is known to cause leaf hyponasty when exogenously applied to plants, does not play a significant role mediating the hyponastic response to low light intensity in Arabidopsis (Millenaar *et al.*, 2009). The same study concluded that auxin perception and PIN3 are necessary for short-term (hours) leaf angle responses to reduced total irradiance (neutral shade). However, although we cannot completely rule out a requirement for auxin action, it is clear that the hyponastic response elicited by blue light attenuation is independent of SAV3 and PIN3. This is most remarkable, particularly because when exposed to low R:FR ratios the hyponastic response is completely absent in *sav3* mutants (Moreno *et al.*, 2009), and is clearly attenuated in *pin3* mutants (Keuskamp *et al.*, 2010a). Therefore, as discussed for the L:P response, we are forced to conclude that the auxin synthesis and transport pathways that are recruited by phyB inactivation are not used to mount responses to BL attenuation, even though the ultimate responses (hyponasty) are very similar.

The mechanism that controls the hyponastic response elicited by cry1 inactivation requires further investigation. Some morphological responses to blue light attenuation have been attributed to hydraulic signals, activated by stomatal closure (Barillot *et al.*, 2010). Blue light promotes stomatal opening though the combined action of cryptochromes and phototropins. It has been shown that *cry1* single and *cry1 cry2* double mutants were more tolerant to water deprivation than wild-type plants, and this effect was correlated with a reduced stomatal sensitivity to blue light (Mao *et al.*, 2005). Further work is needed to test whether the effect of blue light attenuation producing leaf hyponasty is functionally connected with stomatal responses.

### PIF4 and PIF5: integrators for multiple signals in the plant canopy?

A key point of convergence between the phyB and cry1 inactivation signals uncovered by this study was the requirement for the bHLH transcription factors PIF4 and PIF5 (Figure 8). Previous work has shown that PIF4 or PIF5 are required for the normal elongation responses of hypocotyls to light of low R:FR (Lorrain *et al.*, 2008). The role of PIFs in the SAS responses of plants at the rosette stage has been less well characterized (Lorrain *et al.*, 2008), and no previous study has demonstrated the involvement of these transcription factors in photomorphogenic responses activated by specific blue light photoreceptors (Leivar and Quail, 2011). Our experiments show that PIF4 is required for normal L:P and petiole elongation responses, and that both PIF4 and PIF5 need to be present for full expression of the hyponastic response to blue light attenuation (Figure 8). Interestingly, PIF4 was also found to be essential for hyponastic responses induced by high temperatures in Arabidopsis, with no obvious redundancy with other PIFs (Koini *et al.*, 2009).

**Figure 9:**
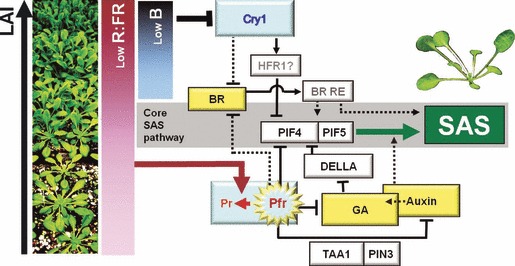
Proposed model for signal integration during shade avoidance syndrome (SAS) responses of Arabidopsis rosettes to increased canopy density (indicated by increased leaf area index, LAI). At low LAIs, only the phyB pathway is triggered in response to far-red radiation reflected from neighboring plants. At high LAIs, when mutual shading among neighbors becomes intense, the reduction in blue light activates the cry1 response pathway, which converges with the phyB pathway at the level of BR, PIF4 and PIF5, thereby boosting the SAS response. The mechanisms and signaling elements (BR-RE: BR-response elements) connecting BR with SAS are not known.

Collectively, these results suggest a model in which PIFs integrate the effects of several environmental and hormonal pathways in the control of leaf angle and morphology in canopies (Figure 9). The mechanisms of interaction between cry1 and PIF proteins need to be investigated in future studies. In the case of phyB, there is direct interaction between PIF and photo-activated phyB in the nucleus, and indirect interactions mediated by the phyB control of GA synthesis and DELLA stability (Feng *et al.*, 2008; Lorrain *et al.*, 2008; de Lucas *et al.*, 2008; Kami *et al.*, 2010). There is no evidence of cry-induced degradation of PIFs, or of physical interactions between crys and PIFs (Leivar and Quail, 2011), and our data show that blue light attenuation treatments do no result in increased DELLA turnover (Figure 4). A potential mechanism whereby blue light attenuation activates PIF4 and PIF5 might involve a related bHLH factor, HFR1 (for long hypocotyl in far-red light; Fairchild *et al.*, 2000). Previous work has established that HFR1 is a positively acting component in cry1 signaling (Duek and Fankhauser, 2003), and recent studies demonstrate that HFR1 directly inhibits the action of growth-promoting PIFs by forming transcriptionally inactive, non-DNA binding heterodimers (Hornitschek *et al.*, 2009).

## Conclusions

Understanding how plants detect their neighbors is a requisite for the intelligent modification of plant responses to population density in order to increase crop yield per unit area. The data reported in this paper show that mutations that knock down key players involved in phyB signaling (*phyB* and *sav3*) do not eliminate the SAS responses of Arabidopsis plants to increased canopy density. This conserved SAS phenotype in mutants impaired in phyB signaling indicates the presence of additional signaling circuits. Communication theory maintains that redundancy is a requirement for the reliable transmission and processing of information in noisy environments (Lesne, 2008). Our results suggest that plants use low R:FR and blue light as partially redundant signals of the proximity of competitors. Low R:FR is the main signal at low canopy LAIs, where plants can sense FR radiation reflected from neighbors, and both low R:FR and reduced blue light irradiance become important indicators of competition at high LAIs (Ballaré, 1999). Our experiments demonstrate that these signals, perceived by phyB and cry1, respectively, also activate separate signaling networks that show limited overlap with regard to the molecular players involved (Figure 9). Parallel control pathways frequently converge in major regulatory nodes, sometimes characterized as phenotypic capacitors (Levy and Siegal, 2008), which are responsible for ensuring that a robust, adaptive phenotype is produced in response to a diverse array of input signals. Our data suggest that PIF4 and PIF5 are critical hubs in the core SAS pathway, which integrate information from the principal light signaling routes that control adaptive plasticity in plant canopies.

## Experimental procedures

### Plant material

*Arabidopsis thaliana* (L.) Heynh accessions Columbia (Col-0) and Landsberg *erecta* (L*er*) were used as the wild-type controls. The mutants *phyB-9* (Reed *et al.*, 1993), *sav3-2* (Tao *et al.*, 2008), *pin3-3* (Friml *et al.*, 2002), quintuple *yucca yuc3,5,7,8,9* (donated by Y. Zhao, University of California at San Diego), *bri1-301* (weak allele of *bri1*; donated by S. Trupkin), *det2-1* (Chory *et al.*, 1991), *cry1-301* (Mockler *et al.*, 1999), *cry2-1* (Guo *et al.*, 1998), *cry1 cry2* (*cry1-hy4-b104 cry2-1*; Buchovsky *et al.*, 2008), *phot1-5 phot2-1* (both donated by H. Boccalandro), *pif4-101* and *pif4pif5* (de Lucas *et al.*, 2008) were all in the Col background. The GA-insensitive gain-of-function *gai1* mutant (Koorneef *et al.*, 1985) and *gai-t6 rga-t2 rgl1-1 rgl3-1 rgl2-1*/SGT625-5 quintuple *della* (CS 16298-ABRC) mutants were all in the L*er* background.

### Growth conditions

For all experiments, seeds were sown onto solid agar (0.8%, w/v) in Petri dishes and stratified at 4°C in darkness for 48 h. They were then irradiated for 1 h with red light and transferred to white light (120 μmol m^−2^ s^−1^). Seedlings were grown for 5 or 6 days until they were transplanted to individual pots or trays with a 1 : 1 : 1 vermiculite : perlite : peat mixture. For the density experiments, seedlings were transplanted to 20 × 16 × 4.5 cm trays in monocultures of four densities: 394, 701, 1315 and 2456 plants per m^2^ (9, 20, 42 and 81 plants per tray in a square planting arrangement), and placed in glasshouse conditions. For each genotype tested (Col-0, *phyB-9* and *sav3-2*) there were four replicates per density. Plants were allowed to compete above and below ground. For the *Lolium* canopy shade experiments, Arabidopsis Col-0 and *sav3-2* seedlings were transplanted to individual plastic pots (0.2 L) and grown in a glasshouse under full sunlight, until they were 21 days old. For the non-shading grass neighbor set-up, the plants were placed on the north side of a thick *L. multiflorum* canopy (the experiment was performed in the southern hemisphere; therefore, Arabidopsis seedlings were not shaded by the grass leaves). To produce the two levels of shading (partial shade and total shade), the plants were placed under the *L. multiflorum* canopy at two different heights, which resulted in the attenuation of photosynthetically active radiation (PAR) of 75 or 92%, respectively. All the glasshouse experiments were carried out in the autumn, under short photoperiods (10–11 h per day); daily temperatures fluctuated between 11 and 25°C. Peak levels of PAR were between 700 and 1000 μmol m^−2^ s^−1^. Trays and individual pots were watered daily with tap water until the appearance of the sixth rosette leaf, and then twice a week with a solution (1 ×) of HAKAPHOS Rojo 18-18-18 (Compo).

For the light manipulation experiments, seedlings were transplanted to soil in individual pots and placed in a growth chamber (photoperiod = 10 h; PAR = 120 μmol m^−2^ s^−1^, standard fluorescent bulbs; temperature = 21°C). The plants were between 14 and 16 days post germination (with between five and seven true leaves) at the start of the light manipulation experiment.

### Light treatments and measurements

In the growth chamber, blue light attenuation was achieved with a yellow filter (Code 101; Lee Filters USA, http://www.leefilters.com/); a pink filter (Lee Filters Code 794) was used to attenuate PAR with minimal reduction of the blue light component, and a clear film (clear polyester 0.75 mm; Oeste Aislante, http://www.oesteaislante.com.ar/) was used as a control (see spectral scans in Figure S5). Light measurements were taken with an SKL 904/I SpectroSense2 meter fitted with an SKR 1850 four-channel sensor (Skye Instruments, http://www.skyeinstruments.com). Light spectra were scanned with a USB4000-UV-VIS spectrometer, pre-configured with a DET4-200-850 detector and a QP600-2-SR optical fiber (Ocean Optics, Inc., http://www.oceanoptics.com), and processed with spectrasuite (http://www.oceanoptics.com/Products/spectrasuite.asp).

### Measurements of leaf angle and leaf morphology

In the light attenuation experiments, plants were measured and photographed at the end of the photoperiod of day 7, counted from the initiation of the light treatments, unless otherwise stated. Because leaf inclination in Arabidopsis displays diurnal variation (Mullen *et al.*, 2006), plants of all genotypes and light treatments were imaged at the same time of the day. Leaf blade angle relative to the horizontal plane was measured in the tallest (petiolated) leaf of the rosette with a protractor. Petiole and lamina lengths were measured with a digital caliper in the fifth or sixth leaf, which sometimes corresponded to the same leaf used to measure the leaf angle.

In the density experiment, plant morphological measurements were taken every week. Measurements taken 25 and 45 days after germination were used to construct Figure 1. Because the initial rate of leaf area expansion varied among genotypes, we used relative canopy cover (instead of the actual plant densities) to compare the different genotypes with regard to their morphological responses to crowding, essentially as described by Ballaré and Scopel (1997). In the canopy shading experiment, plants were measured and photographed as indicated for the light attenuation experiments.

### DELLA abundance

The *RGA* coding sequence was amplified from Col-0 genomic DNA with the following primers (*RGA-B2R*, GGGGACAGCTTTCTTGTACAAAGTGGCTATGAAGAGAGATCATCAC; *RGA-B3wSTOP*, GGGGACAACTTTGTATAATAAAGTTGCTCAGTACGCCGCCGTCGA) and cloned into *pDONR-P2RP3* using gateway recombination (Invitrogen, http://www.invitrogen.com). *mCITRINE* was amplified with the following primers (*mCITRINE-B1wKOZAK*, GGGGACAAGTTTGTACAAAAAAGCAGGCTTAACCATGGTGAGCAAGGGCGAG; *mCITRINE-B2noSTOP*, GGGGACCACTTTGTACAAGAAAGCTGGGTACTTGTACAGCTCGTCCATGCC) and cloned into *pDONR221* using gateway recombination as well (Invitrogen). The final destination vector was obtained with a three-fragment recombination system using *pUBQ10/pDONRP4P1R* (Jaillais *et al.*, 2011), *mCITRINE/pDONR221* and *RGA/pDONRP2RP3* entry vectors and *pB7m34GW* as a destination vector (Karimi *et al.*, 2007). The *pUBQ10::mCITRINE-RGA* was transformed into Col-0 and selected using its glyfosinate (Basta) resistance. Confocal microscopy was carried out with a Leica SP/2 inverted microscope (Leica, http://www.leica.com). The same confocal settings were used in all conditions, and each image is representative of three independent experiments (eight plants per genotype and experiment).

### Pharmacological experiments

To investigate the involvement of auxin polar transport, intact Col-0 rosette plants were sprayed at 15 days after germination with an aqueous solution of the polar auxin transport inhibitor NPA (Sigma-Aldrich, http://www.sigmaaldrich.com). NPA stock solutions (1000 ×) were prepared in DMSO. Working solutions at final concentrations of 0.5, 5 and 50 μm NPA and 0.1% Tween 20 were sprayed onto the plants. Control plants were sprayed with solutions containing equivalent quantities of DMSO and Tween 20, without NPA. After applying NPA, plants were immediately placed under the light treatments. Morphological data were obtained 4 days after NPA application.

### Statistical analyses

Experimental data were analysed using infostat 1.1 (http://www.infostat.com.ar) by means of factorial analysis of variance (anova). In the figures, we report the significance of the main effect of the light treatments. If the light effect varied with genotype (i.e. in cases of a significant light × genotype interactions; L × G), treatment means were further separated using Tukey’s comparisons. In this case, significant differences between treatment means are indicated by different letters within a given genotype.

## SUPPORTING INFORMATION

**Figure S1.** Shade avoidance syndrome (SAS) responses to blue light attenuation are conserved in quintuple *yucca* mutants.

**Figure S2.** Effect of NPA on leaf angle, petiole length and lamina/petiole length ratio (L:P) in response to low blue light treatment.

**Figure S3.** GA treatment triggered rapid mCITRINE-RGA turnover.

**Figure S4.** Representative photographs of the phenotype of the *det2-1* mutant under the light conditions used in this study (after 7 days of treatment).

**Figure S5.** Characteristics of the light environment in the canopy experiments and spectral photon distribution under the light treatments used in the filtration experiments.

Please note: As a service to our authors and readers, this journal provides supporting information supplied by the authors. Such materials are peer-reviewed and may be re-organised for online delivery, but are not copy-edited or typeset. Technical support issues arising from supporting information (other than missing files) should be addressed to the authors.

## References

[b1] Aphalo PJ, Ballaré CL (1995). On the importance of information-acquiring systems in plant-plant interactions. Funct. Ecol.

[b2] Ballaré CL (1999). Keeping up with the neighbours: phytochrome sensing and other signalling mechanisms. Trends Plant Sci.

[b3] Ballaré CL (2009). Illuminated behaviour: phytochrome as a key regulator of light foraging and plant anti-herbivore defence. Plant Cell Environ.

[b4] Ballaré CL, Scopel AL (1997). Phytocrome signalling in plant canopies. Testing its population-level consequences using photoreceptor mutants of Arabidopsis. Funct. Ecol.

[b5] Ballaré CL, Scopel AL, Sanchez RA (1991). Photocontrol of stem elongation in plant neighbourhoods: effects of photon fluence rate under natural conditions of radiation. Plant Cell Environ.

[b6] Ballaré CL, Scopel AL, Radosevich SR, Kendrick RE (1992). Phytochrome-mediated phototropism in de-etiolated seedlings. Plant Physiol.

[b7] Barillot R, Frak E, Combes D, Durand JL, Escobar-Gutiérrez AJ (2010). What determines the complex kinetics of stomatal conductance under blueless PAR in *Festuca arundinacea*? Subsequent effects on leaf transpiration. J. Expt. Bot.

[b8] Benschop JJ, Millenaar FF, Smeets ME, Van Zanten M, Voesenek LACJ, Peeters AJM (2007). Abscisic acid antagonizes ethylene-induced hyponastic growth in Arabidopsis. Plant Physiol.

[b9] Briggs WR, Christie JM (2002). Phototropins 1 and 2: versatile plant blue-light receptors. Trends Plant Sci.

[b10] Buchovsky AS, Strasser B, Cerdán PD, Casal JJ (2008). Suppression of pleiotropic effects of functional CRYPTOCHROME genes by TERMINAL FLOWER 1. Genetics.

[b11] Cashmore AR, Jarillo JA, Wu Y-J, Liu D (1999). Cryptochromes: blue light receptors for plants and animals. Science.

[b12] Chory J, Nagpal P, Peto CA (1991). Phenotypic and genetic analysis of *det2*, a new mutant that affects light-regulated seedling development in Arabidopsis. Plant Cell.

[b13] De Kroon H, Visser EJW, Huber H, Mommer L, Hutchings MJ (2009). A modular concept of plant foraging behaviour: the interplay between local responses and systemic control. Plant Cell Environ.

[b14] Djakovic-Petrovic T, Wit Md, Voesenek LACJ, Pierik R (2007). DELLA protein function in growth responses to canopy signals. Plant J.

[b15] Dorn LA, Pyle EH, Schmitt J (2000). Plasticity to light cues and resources in *Arabidopsis thaliana*: testing for adaptive value and costs. Evolution.

[b16] Duek PD, Fankhauser C (2003). HFR1, a putative bHLH transcription factor, mediates both phytochrome A and cryptochrome signalling. Plant J.

[b17] Fairchild CD, Schumaker MA, Quail PH (2000). HFR1 encodes an atypical bHLH protein that acts in phytochrome A signal transduction. Genes Dev.

[b18] Feng SH, Martinez C, Gusmaroli G (2008). Coordinated regulation of *Arabidopsis thaliana* development by light and gibberellins. Nature.

[b19] Folta KM, Spalding EP (2001). Unexpected roles for cryptochrome 2 and phototropin revealed by high-resolution analysis of blue light-mediated hypocotyl growth inhibition. Plant J.

[b20] Franklin KA (2008). Shade avoidance. New Phytol.

[b21] Frigerio M, Alabadí D, Pérez-Gómez J, García-Cárcel L, Phillips AL, Hedden P, Blázquez MA (2006). Transcriptional regulation of gibberellin metabolism genes by auxin signaling in Arabidopsis. Plant Physiol.

[b22] Friml J, Wiśniewska J, Benková E, Mendgen K, Palme K (2002). Lateral relocation of auxin efflux regulator PIN3 mediates tropism in Arabidopsis. Nature.

[b23] Guo H, Yang H, Mockler TC, Lin C (1998). Regulation of flowering time by Arabidopsis photoreceptors. Science.

[b24] Harberd NP, Belfield E, Yasumura Y (2009). The Angiosperm gibberellin-GID1-DELLA growth regulatory mechanism: how an “inhibitor of an inhibitor” enables flexible response to fluctuating environments. Plant Cell.

[b25] Hornitschek P, Lorrain S, Zoete V, Michielin O, Fankhauser C (2009). Inhibition of the shade avoidance response by formation of non-DNA binding bHLH heterodimers. EMBO J.

[b26] Jaillais Y, Chory J (2010). Unraveling the paradoxes of plant hormone signaling integration. Nat. Struct. Mol. Biol.

[b27] Jaillais Y, Hothorn M, Belkhadir Y, Dabi T, Nimchuk ZL, Meyerowitz EM, Chory J (2011). Tyrosine phosphorylation controls brassinosteroid receptor activation by triggering membrane release of its kinase inhibiton. Genes Dev.

[b28] Kami C, Lorrain S, Hornitschek P, Fankhauser C, Timmermans MCP (2010). Light-regulated plant growth and development. Current Topics in Developmental Biology.

[b29] Karimi M, Depicker A, Hilson P (2007). Recombinational cloning with plant gateway vectors. Plant Physiol.

[b30] Keuskamp DH, Pollmann S, Voesenek LACJ, Peeters AJM, Pierik R (2010a). Auxin transport through PIN-FORMED 3 (PIN3) controls shade avoidance and fitness during competition. Proc. Natl Acad. Sci. USA.

[b31] Keuskamp DH, Sasidharan R, Pierik R (2010b). Physiological regulation and functional significance of shade avoidance responses to neighbors. Plant Signal Behav.

[b32] Keuskamp DH, Sasidharan R, Vos I, Peeters AJM, Voesenek LACJ, Pierik R (2011). Blue light-mediated shade avoidance requires combined auxin and brassinosteroid action in Arabidopsis seedlings. Plant J.

[b33] Koini MA, Alvey L, Allen T, Tilley CA, Harberd NP, Whitelam GC, Franklin KA (2009). High temperature-mediated adaptations in plant architecture require the bHLH transcription factor PIF4. Curr. Biol.

[b34] Koorneef M, Elgersma A, Hanhart CJ, van Loenen-Martinet EP, van Rijn L, Zeevaart JAD (1985). A gibberellin insensitive mutant of *Arabidopsis thaliana*. Physiol. Plant.

[b35] Kozuka T, Horiguchi G, Kim G-T, Ohgishi M, Sakai T, Tsukaya H (2005). The different growth responses of the *Arabidopsis thaliana* leaf blade and the petiole during shade avoidance are regulated by photoreceptors and sugar. Plant Cell Physiol.

[b36] Kozuka T, Kobayashi J, Horiguchi G, Demura T, Sakakibara H, Tsukaya H, Nagatani A (2010). Involvement of auxin and brassinosteroid in the regulation of petiole elongation under the shade. Plant Physiol.

[b37] Leivar P, Quail PH (2011). PIFs: pivotal components in a cellular signaling hub. Trends Plant Sci.

[b38] Lesne A (2008). Robustness: confronting lessons from physics and biology. Biol. Rev.

[b39] Levy SF, Siegal ML (2008). Network hubs buffer environmental variation in Saccharomyces cerevisiae. PLoS Biol.

[b40] Lorrain S, Allen T, Duek PD, Whitelam GC, Fankhauser C (2008). Phytochrome-mediated inhibition of shade avoidance involves degradation of growth-promoting bHLH transcription factors. Plant J.

[b41] de Lucas M, Daviere JM, Rodriguez-Falcon M, Pontin M, Iglesias-Pedraz JM, Lorrain S, Fankhauser C, Blazquez MA, Titarenko E, Prat S (2008). A molecular framework for light and gibberellin control of cell elongation. Nature.

[b42] Luccioni LG, Oliverio KA, Yanovsky MJ, Boccalandro HE, Casal JJ (2002). Brassinosteroid mutants uncover fine tuning of phytochrome signaling. Plant Physiol.

[b43] Luo XM, Lin WH, Zhu S (2010). Integration of light- and brassinosteroid-signaling pathways by a GATA transcription factor in Arabidopsis. Dev Cell.

[b44] Mao J, Zhang YC, Sang Y, Li QH, Yang HQ (2005). A role for Arabidopsis cryptochromes and COP1 in the regulation of stomatal opening. Proc. Natl Acad. Sci. USA.

[b45] Martínez-García JF, Galstyan A, Salla-Martret M, Cifuentes-Esquivel N, Gallemí M, Bou-Torrent J, Jean-Claude K, Michel D (2010). Regulatory Components of Shade Avoidance Syndrome.

[b46] Millenaar FF, Van Zanten M, Cox MCH, Pierik R, Voesenek LACJ, Peeters AJM (2009). Differential petiole growth in *Arabidopsis thaliana*: photocontrol and hormonal regulation. New Phytol.

[b47] Mockler TC, Guo H, Yang H, Duong H, Lin C (1999). Antagonistic actions of Arabidopsis cryptochromes and phytochrome B in the regulation of floral induction. Development.

[b48] Moreno JE, Tao Y, Chory J, Ballaré CL (2009). Ecological modulation of plant defense via phytochrome control of jasmonate sensitivity. Proc. Natl Acad. Sci. USA.

[b49] Mullen JL, Weinig C, Hangarter RP (2006). Shade avoidance and the regulation of leaf inclination in Arabidopsis. Plant Cell Environ.

[b50] Neff MM, Nguyen SM, Malancharuvil EJ (1999). BAS1: a gene regulating brassinosteroid levels and light responsiveness in *Arabidopsis*. Proc. Natl Acad. Sci. USA.

[b51] Novoplansky A (2009). Picking battles wisely: plant behaviour under competition. Plant Cell Environ.

[b52] Okada K, Ueda J, Komaki MK, Bell CJ, Shimura Y (1991). Requirement of the auxin polar transport system in early stages of arabidopsis floral bud formation. Plant Cell.

[b53] Pierik R, Whitelam GC, Voesenek LACJ, de Kroon H, Visser EJW (2004). Canopy studies on ethylene-insensitive tobacco identify ethylene as a novel element in blue light and plant-plant signalling. Plant J.

[b54] Pierik R, Djakovic-Petrovic T, Keuskamp DH, de Wit M, Voesenek LACJ (2009). Auxin and ethylene regulate elongation responses to neighbor proximity signals independent of gibberellin and DELLA proteins in Arabidopsis. Plant Physiol.

[b55] Reed JW, Nagpal P, Poole DS, Furuya M, Chory J (1993). Mutations in the gene for the red/far-red light receptor phytochrome B alter cell elongation and physiological responses throughout arabidopsis development. Plant Cell.

[b56] Sellaro R, Crepy M, Trupkin SA, Karayekov E, Buchovsky AS, Rossi C, Casal JJ (2010). Cryptochrome as a sensor of the blue/green ratio of natural radiation in Arabidopsis. Plant Physiol.

[b57] Smith H (1982). Light quality, photoperception, and plant strategy. Annu. Rev. Plant Physiol.

[b58] Sultan SE (2010). Plant developmental responses to the environment: eco-devo insights. Curr. Opin. Plant Biol.

[b59] Szekeres M, Németh K, Koncz-Kálmán Z, Mathur J, Kauschmann A, Altmann T, Rédei GP, Nagy F, Schell J, Koncz C (1996). Brassinosteroids rescue the deficiency of CYP90, a cytochrome P450, controlling cell elongation and de-etiolation in Arabidopsis. Cell.

[b60] Takemiya A, Inoue S-i, Doi M, Kinoshita T, Shimazaki K-i (2005). Phototropins promote plant growth in response to blue light in low light environments. Plant Cell.

[b61] Tao Y, Ferrer JL, Ljung K (2008). Rapid synthesis of auxin via a new tryptophan-dependent pathway is required for shade avoidance in plants. Cell.

[b62] Vandenbussche F, Pierik R, Millenaar FF, Voesenek LACJ, Van Der Straeten D (2005). Reaching out of the shade. Curr. Opin. Plant Biol.

[b63] van Zanten M, Pons TL, Janssen JAM, Voesenek LACJ, Peeters AJM (2010). On the Relevance and Control of Leaf Angle. Critical Rev. Plant Sci.

[b64] Zhao Y, Christensen SK, Fankhauser C, Cashman JR, Cohen JD, Weigel D, Chory J (2001). A role for flavin monooxygenase-like enzymes in auxin biosynthesis. Science.

