# Supplementary figures and images for: Cryptochrome 1 and phytochrome B control shade-avoidance responses in Arabidopsis via partially independent hormonal cascades
Source: Plant J. 2011 May 25;67(2):195–207. doi: 10.1111/j.1365-313X.2011.04598.x (PMC3135679; doi:10.1111/j.1365-313X.2011.04598.x)

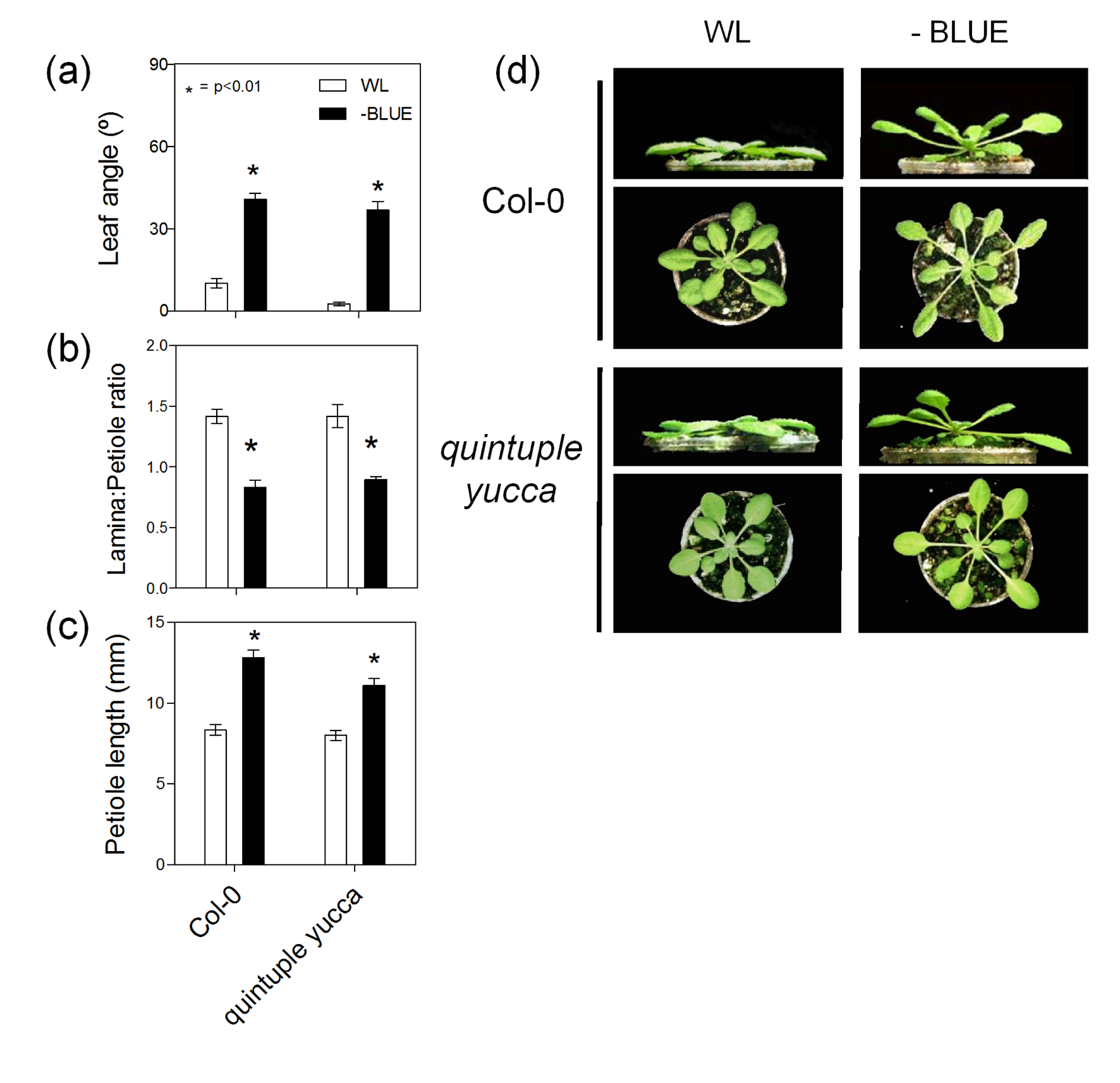

Supplement: Supplementary file 1 [file tpj0067-0195-SD1.tif]

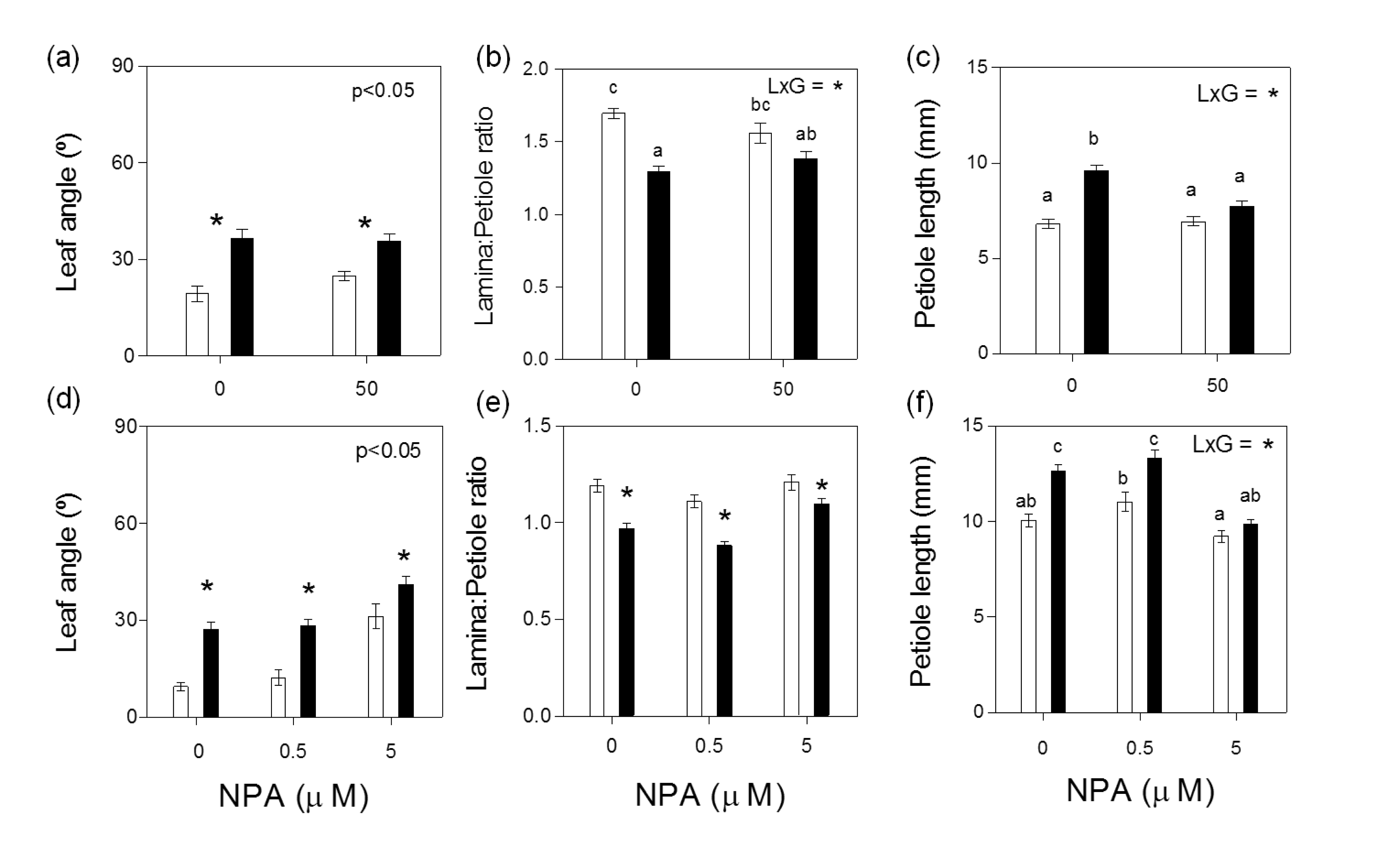

Supplement: Supplementary file 2 [file tpj0067-0195-SD2.tif]

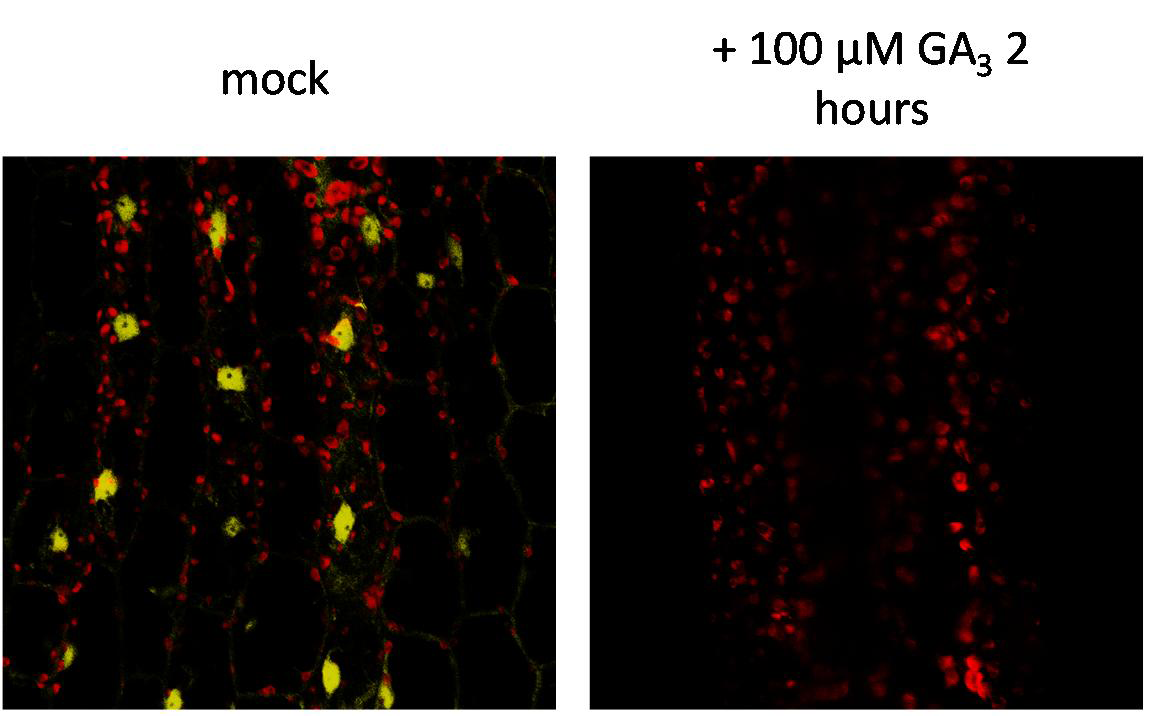

Supplement: Supplementary file 3 [file tpj0067-0195-SD3.tif]

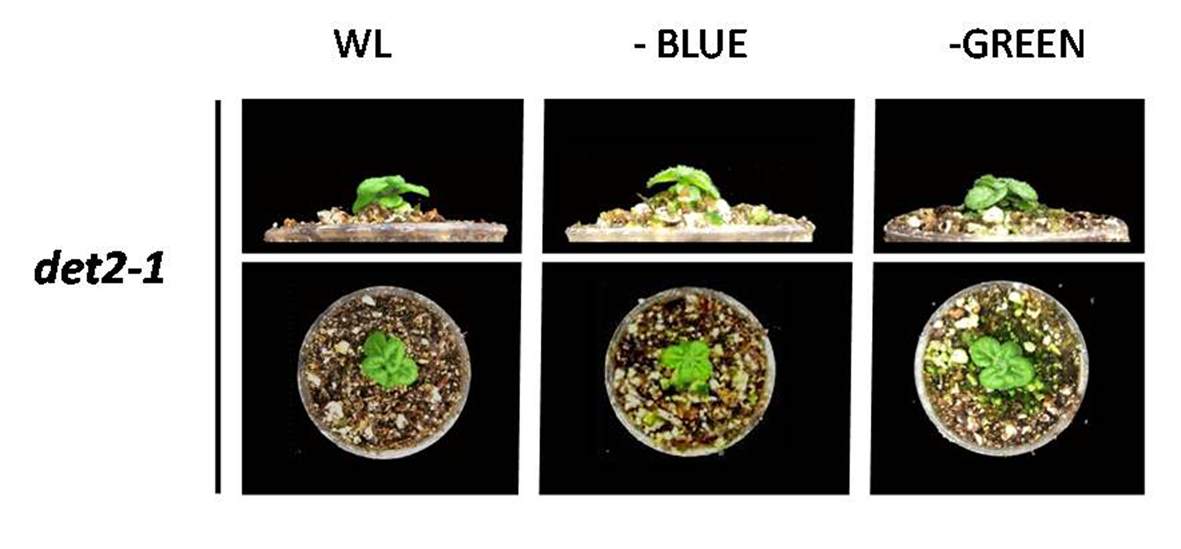

Supplement: Supplementary file 4 [file tpj0067-0195-SD4.tif]

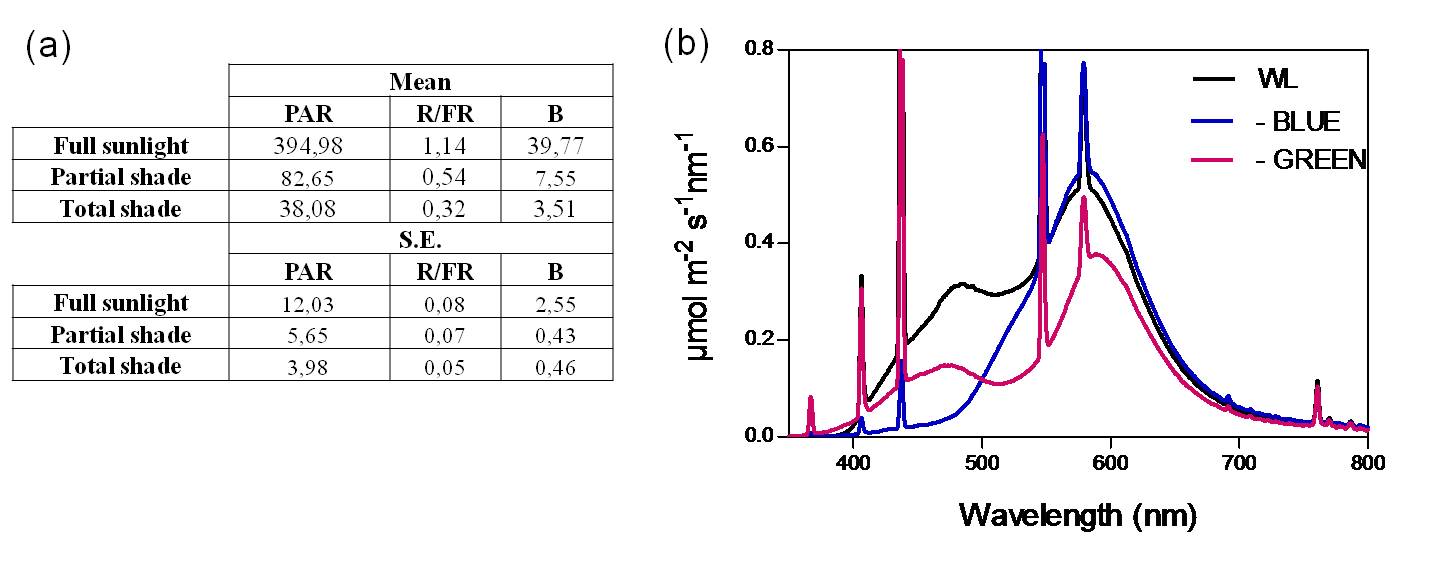

Supplement: Supplementary file 5 [file tpj0067-0195-SD5.jpg]
