# Supplementary material for: Cryptochrome 1 and phytochrome B control shade-avoidance responses in Arabidopsis via partially independent hormonal cascades
Source: Plant J. 2011 May 25;67(2):195–207. doi: 10.1111/j.1365-313X.2011.04598.x (PMC3135679; doi:10.1111/j.1365-313X.2011.04598.x)
Supplement: Supplementary file 6 [file tpj0067-0195-SD6.doc]

**Supplementary material for Keller *et al.***

**Figure S1.** SAS responses to B light attenuation are conserved in quintuple *yucca* mutants. Plants were grown under white light (WL) or WL filtered through a yellow filter (-BLUE). Morphological measurements and photographs were taken after 7 d of treatment. Thin bars indicate ±1 S.E (n=10-12 individual plants). Asterisks indicate significant effects of the light treatments at the indicated P value.(a-c) Comparison of morphological responses between Col-0 and quintuple *yucca* mutants.(d) Representative photographs of the plants after exposure to the indicated light treatments.

**Figure S2**. Effect of NPA on leaf angle, petiole length and L:P ratio of WT Columbia-0 plants in response to low blue treatment. NPA was sprayed onto intact 14-d rosette plants immediately before the beginning of the light treatments. After the NPA treatment, the plants were grown under white light (WL, white bars) or WL filtered through a yellow filter (-BLUE, black bars). Morphological measurements and photographs were taken after 4 d of treatment. Thin bars indicate ±1 S.E. (n=10-12 individual plants). Asterisks indicate significant effects of the light treatments at the indicated P value. Different letters indicate significant differences between means in cases in which the light x genotype interaction term (LxG) was significant.(a-c) NPA effect at high (50 µM) doses. This NPA treatment caused considerably growth inhibition and leaf curling, bud did not prevent the hyponastic response.(d-f) NPA effect at low doses (≤ 5 µM). Notice that these low NPA doses had *per se* a positive effect promoting a hyponastic response.

**Figure S3.** GA treatment triggered rapid mCITRINE-RGA turnover in the *pUBQ10::mCITRINE-RGA* line.

**Figure S4.** Representative photographs of the phenotype of the *det2-1* mutant under the attenuated blue light conditions used in this study. Photographs were taken after 7 d of treatment.

**Figure S5**. Irradiances and spectral photon distributions under the light treatments used in the canopy and filtration experiments, respectively.

1. Mean and Standard Error values for the PAR and B irradiances (µmoles.m^-2^.s^-1^) and the R:FR ratio characteristic of light environments in the glasshouse canopy experiments shown in Figure 2.
2. Spectral photon distributions under the light treatments used in the growth chamber filtration experiments.
